# Risk factors for invasive meningococcal disease: a retrospective analysis of the French national public health insurance database

**DOI:** 10.1080/21645515.2020.1849518

**Published:** 2021-01-15

**Authors:** Muhamed-Kheir Taha, Catherine Weil-Olivier, Stéphane Bouée, Corinne Emery, Gaëlle Nachbaur, Céline Pribil, Véronique Loncle-Provot

**Affiliations:** a Invasive Bacterial Infections Unit, Institut Pasteur, Paris, France; b Service de Pédiatrie, Université de Paris VII Diderot, Paris, France; c RWE Department, CEMKA-EVAL, Bourg-la-Reine, France; d Vaccine Medical Department, GSK, Rueil-Malmaison, France

**Keywords:** Invasive meningococcal disease, risk factors, immunodeficiency, social deprivation, case-control study; respiratory tract infection

## Abstract

Vaccination of at-risk populations against *Neisseria meningitidis* is an important strategy to prevent invasive meningococcal disease (IMD). The objective of this study was to characterize preexisting risk factors in patients with IMD and to compare their relative importance. This case-control analysis was performed in the French national public health insurance database (*SNDS*). Cases consisted of all people hospitalized for IMD in France over a six-year period (2012–2017). Controls were matched by age, gender, and district of residence. Medical risk factors were identified from ICD-10 codes in the *SNDS*. Socioeconomic risk factors studied were low household income and social deprivation of the municipality of residence. Associations of these risk factors with hospitalization for IMD were quantified as odds ratios (ORs) between cases and controls with their 95% confidence intervals (95%CI). The medical risk factors showing the most robust associations were congenital immunodeficiency (OR: 39.1 [95%CI: 5.1–299], acquired immunodeficiency (10.3 [4.5–24.0]) and asplenia/hyposplenia (6.7 [3.7–14.7]). In addition, certain chronic medical conditions, such as autoimmune disorders (5.4 [2.5–11.8]), hemophilia (4.7 [1.8–12.2]) and severe chronic respiratory disorders (4.3 [3.1–6.2]) were also strongly associated, as was low household income (1.68 [1.49–1.80]). In conclusion, this study has documented potential risk factors associated with hospitalization for IMD in a large and comprehensive sample of individuals with IMD in France. Several of the risk factors identified may help identify groups who could benefit from targeted prevention measures (such as vaccination) in order to reduce the burden of IMD.

## Introduction

Invasive meningococcal disease (IMD) is a systemic infection caused by the Gram-negative capsulated bacterium *Neisseria meningitidis* (Nm).^1^ Although this pathogen commonly colonizes the human nasopharynx (and to a lesser extent, the urogenital tract and anal canal) and can be isolated from around 10% of individuals, invasive disease is rare.^1-3^ IMD is a serious, rapidly progressing condition, associated with life-threatening sepsis, with a case fatality rate of 6–8% in spite of appropriate treatment, and a high associated risk of severe sequelae.^1,4^ Amputation due to meningococcemic sepsis (purpura fulminans) may be necessary,^5^ and major permanent neurological or sensory sequelae are reported in up to 20% of cases.^6,7^ In Europe, the incidence rate of IMD is around 1 case per 100,000 individuals, although this rate oscillates over time and differs between countries.^8^ Different serogroups of Nm have been described which differ in the composition of their capsular polysaccharides.^9^ Twelve such serogroups have been identified, of which the most frequent in Europe is serogroup B, accounting for around half of identified cases, followed by serogroups C, Y and W.^10^

Due to persistent high morbidity and mortality of IMD, vaccination has been introduced in many countries. Conjugate meningococcal vaccines against the C serogroup (MenC vaccines) were the first to be developed. Systematic vaccination of infants with MenC was introduced in the United Kingdom in 1999, and this was followed by the development of multivalent conjugate vaccines against multiple serogroups (MenACWY), introduced in 2010 in the USA. These vaccination programs showed a significant impact on the IMD burden, as well as on the extent of asymptomatic carriage.^8,11^ Recently, two recombinant protein MenB vaccines have been licensed and introduced in a growing number of countries.^11^ National vaccination policies vary considerably between countries with respect to obligations, recommendations, and reimbursement.^12^ Many countries only recommend vaccination in groups of individuals who are considered to be at high risk. Nonetheless, all recommendations agree on the requirement for vaccination of at-risk individuals, although the definition of at-risk groups varies considerably between countries (Supplementary Table 1). This variation may be explained at least in part by the fact that the relative importance of these risk factors in the population as a whole remains poorly documented.

To date, IMD risk factors have generally been investigated in relatively small case series, often following large IMD outbreaks populations, for example in university students.^13-15^ For rare diseases such as IMD, it is difficult to perform general population-based studies to identify and quantify risk factors in an empirical way. However, the availability of comprehensive medical information databases provides an opportunity to collect and compare data for much larger numbers of IMD cases, and to compare the importance of clinical risk factors in these individuals. In France, the *SNDS* (*Système National d’Information Inter-régimes de l’Assurance Maladie*) database, the healthcare delivery and reimbursement database of the French national public health insurance system, covers around 99% of the French population (~66 million individuals).^16^ This database has been used successfully for epidemiological studies of rare diseases to address questions that cannot easily be answered by clinical studies.^17-20^ The objective of the present case-control study was to characterize preexisting risk factors in all cases of people hospitalized for IMD in France over six years from 2012 to 2017, identified using the *SNDS* database.

## Methods

This observational case-control study was conducted in the *SNDS*. Cases comprised all incident cases of acute IMD resulting in hospitalization in France between January 1st, 2012, and December 31th, 2017.

In the SNDS database, each patient is identified by a unique identifier, which allows information to be linked for individual patients from all the constituent data sets that make up the SNDS database. The constituent data sets exploited in this study related to hospitalizations, community care, prescriptions, health insurance status and long-term illness status.

### Identification of cases and controls

Cases were identified from hospitalization records and defined as all individuals hospitalized during the inclusion period of the study with an ICD-10 diagnostic code (A39.0 to A39.9) consistent with a diagnosis of IMD mentioned on the hospital discharge summary. The date of the first hospitalization was taken as the index date.

The SNDS database was also used to identify the controls, since it contains >99% of the French general population, and all individuals in the database are coded identically. Controls were matched to cases for age, gender, and administrative district of residence (département) on the index date of the case, this being the only demographic information available in the SNDS. Three controls were identified at random for each case. Since the objective was to compare the cases to the French general population, it was not considered appropriate to restrict the identification of controls to hospitalized patients, and whether the control was hospitalized or not at the time of the index hospitalization of the matching case was not taken into consideration.

### Data collection

Data were extracted from the *SNDS* database on demographics, health insurance status, comorbidities, and treatments. Demographic information is limited to age, gender, and municipality of residence. The database contains information on whether the insuree is a beneficiary of comprehensive reimbursement for all health expenditure, either due to a chronic disease such as diabetes or heart failure which requires long-term treatment (*affection de longue durée; ALD* status) or due to low income (*couverture maladie universelle complémentaire; CMU-C* status). In the case of *ALD* status, the nature of the chronic disease is specified. Treatments delivered in community pharmacies are documented by name, date, and dose. Treatments delivered in hospital are not usually documented since they are integrated into the unit costs for the hospital stay according to national tariffs. Comorbidities can be documented from hospitalization discharge summaries identified by ICD-10 codes, from ALD status or, in certain cases, from prescription of specific treatments (such as statins for hypercholesterolemia). As patients are identified in the database by a unique identifier, double counting of comorbidities documented from multiple sources can be avoided. Vaccines delivered in pharmacies before the index date were identified by brand name and date of delivery.

### Identification of risk factors

Potential risk factors were evaluated for association with hospitalization for IMD. Three different classes of potential medical risk factors and two potential socioeconomic risk factors were considered. The database codes and algorithms used to identify the medical risk factors are provided in Supplementary Table 2.

The first class ones were known medical risk factors cited in the vaccination recommendations from the public health authorities of the five largest European countries (Germany,^21^ France,^22^ United Kingdom,^23^ Italy,^24^ and Spain;^25^ Supplementary Table 1). Certain risk factors mentioned in the guidelines cannot be identified in the *SNDS* database, for example, hypo-γ-globulinemia (mentioned in the German recommendations), use of high-dose corticosteroids (Italian recommendations) or loss of cerebrospinal fluid (Italian recommendations). For others, proxy variables could be found, although these did not exactly match the original definition of the risk factor. For example, *ALD* status for cancer was used as a proxy for prior chemotherapy use (Italian recommendations) and *ALD* status for chronic renal disease as a proxy for renal insufficiency with a creatinine clearance of <30 ml/min (Italian recommendations), since creatinine clearance is not documented in the *SNDS*. Cases of type 4 toll-like receptor deficiency (Italian recommendations) should have been retrieved under the broader category of congenital immunodeficiency disorders.

A second class included medical variables associated with an elevated risk of IMD described in the scientific literature. These included prematurity,^14,26^ defined by the ICD-10 code P07 used in the *SNDS*, corresponding to ‘Disorders related to short gestation and low birth weight, not elsewhere classified’. Recent influenza infections and other respiratory tract infections have also been suggested to be associated with an elevated risk of IMD.^1,27,28^ For the purposes of this study, recent infections that had occurred in the six months preceding the index IMD episode and requiring hospitalization were evaluated.

Thirdly, chronic diseases present before the index IMD episode identified through eligibility for comprehensive healthcare reimbursement due a severe chronic disease (*ALD* status) were assessed.

Two socioeconomic factors were evaluated: *CMU-C* insurance status was used as a proxy variable for low income. This status is available for all individuals in employment whose income is below a specific threshold. Until young people enter their first salaried employment, their insurance status depends on their parents’ income. Once individuals reach retirement age, they remain eligible for *CMU-C* status if their retirement pension remains below the threshold. Around 10% of the total French population currently benefits from *CMU-C* status. The second socioeconomic factor evaluated was a validated social deprivation index (SDI), used to classify the municipality of residence^29^ into five quintiles, the highest quintile representing the most deprived municipalities.

### Statistical analysis

The strength of association of potential risk factors for IMD was evaluated by comparing their frequency in cases and controls in univariate regression analysis and expressed as an odds ratio [OR] with its 95% confidence intervals (95%CI). Probability values were determined with the χ2 test or Fisher’s exact test as appropriate. The findings are presented graphically as Forest plots. For both socioeconomic factors, a multivariate logistic analysis was performed to determine their relative importance. All statistical analyses were performed using SAS® software, Version 9.4 (Cary, NC, USA).

### Ethics

The study was conducted in accordance with all relevant international and French regulatory requirements. Patient data in the database are anonymized using an irreversible double encryption. Access to the *SNDS* is regulated by a Committee of Expertise for Research, Studies and Evaluations in the field of Health (CEREES), to which the present study protocol was submitted for approval. Since this was a retrospective study of an anonymized database and had no influence on patient care, ethics committee approval was not required. The use of the *SNDS* database for this type of study is regulated by the French national data protection agency (*Commission Nationale de l’Informatique et des Libertés*), who approved the protocol.

## Results

### Study population

Overall, 3,532 individuals were identified as having been hospitalized for IMD between 2012 and 2017 with the diagnostic code of A39.0 to A39.9. The mean age of the study population was 29.7 ± 27.6 years (median 21). Over this six-year period, the annual number of cases remained relatively stable, although the age distribution oscillated somewhat, with a relative decline in the number of cases in the group of those under 25 years of age from 389 cases in 2012 to 288 in 2017 (Supplementary Table 4). The mean age of each annual cohort increased progressively from 27 years in 2012 to 34 years in 2017, and the median age from 19 to 25 years.

**Figure 1.**
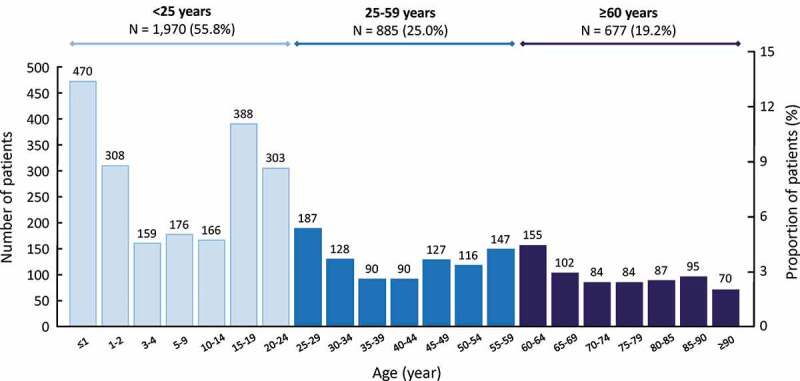
Age distribution of cases of invasive meningococcal disease

The age distribution of the IMD cases is presented in Figure 1. Two peaks in the number of cases were observed, one in infants aged under two years (22.0% of cases) and the second peak in adolescents and young adults aged between 15 and 24 years (19.6%).

Each column represents the total number of cases in the given age group identified during the six-year follow-up period.

Hospitalizations for IMD were generally more frequent in men than in women, except in the oldest age group (over 60 years), in which women accounted for 59% of the cases (Table 1). Overall, 23.2% of cases benefited from full reimbursement of health care consumption due to chronic serious illness at the time of their index hospitalization, principally in the oldest age group. In addition, 16.6% of cases (or their parents), mainly in the group aged under 25 years (20.2%), benefited from full reimbursement due to low incomes (*CMU-C* status) at the time of the index event (Table 1).

**Table 1.** Sociodemographic characteristics of included cases

	Age range			
	<25 years	25–59 years	≥60 years	All ages
Number of cases	1,970	885	677	3,532
Gender				
Male	1,108 (56.2%)	464 (52.4%)	277 (40.9%)	1,849 (52.3%)
Female	862 (43.8%)	421 (47.6%)	400 (59.1%)	1,683 (48.0%)
*ALD* beneficiary	122 (6.4%)	53 (6.0%)	434 (64.1%)	814 (23.2%)
*CMU-C* beneficiary	397 (20.2%)	163 (18.4%)	27 (4.0%)	587 (16.6%)

*ALD: affection de longue durée* (long-term disease status); *CMU-C: couverture maladie universelle complémentaire* (proxy variable for low income).

### Medical risk factors

**Figure 2.**
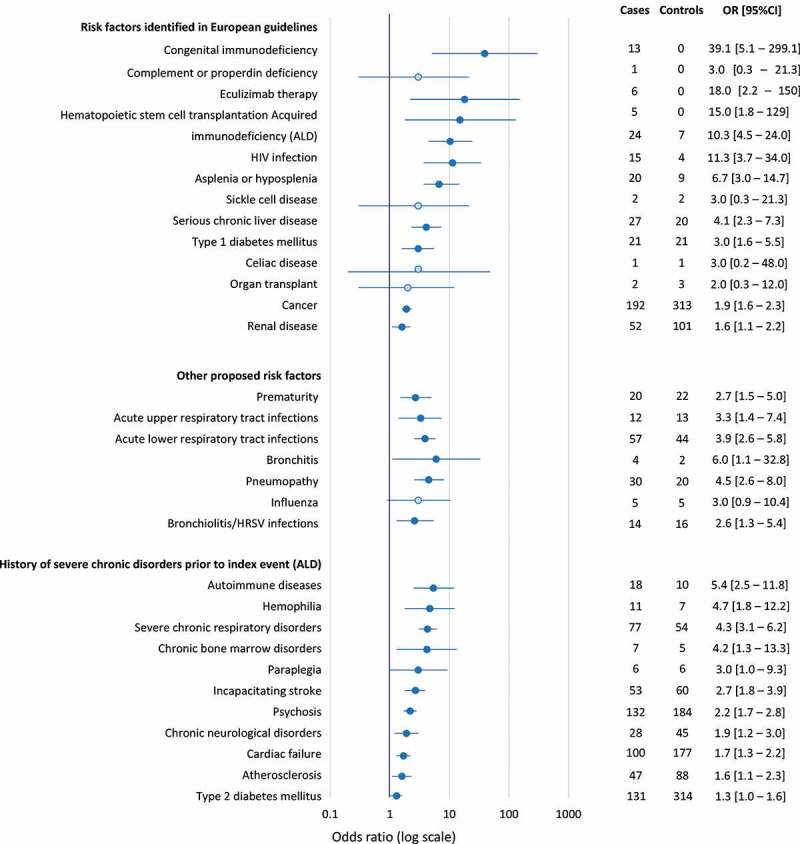
Medical variables associated with hospitalization for invasive meningococcal disease (case-control analysis)

Risk factors were assessed in a case-control analysis involving the 3,532 cases and 10,590 controls, with two cases that could not be matched. The findings are represented in the form of a Forest plot in Figure 2. Regarding risk factors listed in most European vaccination recommendations, asplenia (20 cases), eculizumab treatment (6 cases) and congenital immunodeficiency disorders (13 cases) were documented in relatively few cases only. Nonetheless, all were associated with an increased risk of hospitalization for IMD compared to the controls, with ORs of 6.7, 18.0, and 39.1, respectively (Figure 2). In addition, acquired immunodeficiency disorders in general, and human immunodeficient virus (HIV) infections in particular (mentioned in the German and Italian guidelines), were associated with an increased risk of hospitalization for IMD, as was a history of hemopoietic stem cell transplantation (mentioned in the French guidelines). For other risk factors featuring in the vaccination recommendations, chronic liver disease, type 1 diabetes mellitus, chronic renal disease, and cancer (used as a proxy variable for prior chemotherapy delivery) also showed significant association with hospitalization for IMD. On the other hand, it was not possible to demonstrate such an association for previous solid organ transplantation (mentioned in the Italian guidelines) and a history of celiac disease (mentioned in the British guidelines), as these occurred in too few cases (one or two). Prematurity was also significantly associated with hospitalization for IMD (Figure 2). With regard to acute infections requiring hospitalization in the previous six months, upper respiratory tract infections (as a class), lower respiratory tract infections (as a class), bronchitis, pneumopathy and bronchiolitis (or human respiratory syncytial virus infections) were associated with hospitalization for IMD (Figure 2). Of the 30 individual diseases giving right to *ALD* status (Supplementary Table 4), 13 were associated with an increased risk of hospitalization for IMD (Figure 2). The strongest associations were observed for autoimmune disorders (as a class, including the individual *ALD*s of systemic lupus erythematosus, rheumatoid arthritis and multiple sclerosis), severe chronic respiratory disorders, and hemophilia.

The ratio of cases to controls was 1:3. Darker blue circles indicate risk factors for which the 95% confidence interval (95% CI) of the odds ratio (OR) exclude unity (significant associations) and the lighter blue circles indicate non-significant associations. Risk factors in *italics* are subsets of the higher-level class of risk factors.Autoimmune disease includes multiple sclerosis, systemic lupus erythematosus, Severe spondylarthritis, vasculitis, systemic scleroderma, rheumatoid arthritis, ulcerative colitis and Crohn’s disease. *ALD: affection de longue durée* (long-term disease status); HIV: human immunodeficiency virus; HRSV: human respiratory syncytial virus.

### Socioeconomic risk factors

**Figure 3.**
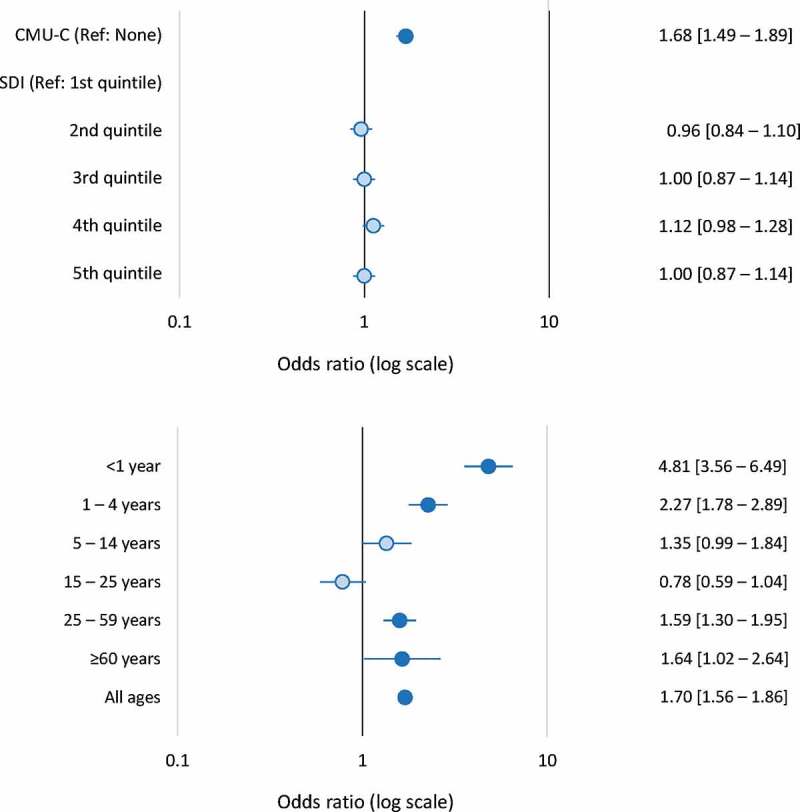
Socioeconomic variables associated with hospitalization for invasive meningococcal disease (case-control analysis)

In the univariate analysis, low income (identified by the proxy variable of *CMU-C* status) and living in a relatively socially deprived community (SDI ≥median value) were both associated with an increased risk of hospitalization for IMD. Overall, the OR for *CMU-C* status compared to no such status was 1.70 [95% CI; 1.56–1.86] and the OR for an SDI above the median value compared to below the median was 1.07 [1.00–1.11]. When these two variables were tested together in a multivariate logistic regression analysis controlling for age, gender, district of residence, and ALD status, only the risk associated with CMU-C status and not that associated with the SDI was retained in the model. The odds ration obtained in the multivariate analysis are presented as a Forest plot in Figure 3(A).

A. Hospitalization for invasive meningococcal disease as a function of *CMU-C* status and of social deprivation index.B. Hospitalization for invasive meningococcal disease as a function of *CMU-C* status and age.The ratio of cases to controls was 1:3. Darker blue circles indicate risk factors for which the 95% confidence interval (95% CI) of the odds ratio (OR) exclude unity (significant associations) and the lighter blue circles indicate non-significant associations.CMU-C: *couverture maladie universelle complémentaire* (proxy variable for low income); SDI: social deprivation index.

The association between *CMU-C* status and risk of hospitalization for IMD was also evaluated as a function of age (Figure 3(B)). In children and young adults, this association was inversely related to age, with the highest risk being observed in infants under the age of one year (OR: 4.81 [3.56–6.49]). For children of school age (5–14 years) or young adults <25 years old, no increased risk associated with the *CMU-C* status of the parents was observed. However, over the age of 25 years, when individuals would be eligible for *CMU-C* status in their own right, the increased risk associated with *CMU-C* status reappeared (Figure 3(B)).

### Vaccination status

Prior to the index hospitalization, 212 cases (6.0%) of the cases had been vaccinated against *N. meningitidis*, of whom 208 were under 25 years of age. Cases principally received a MenC vaccine (209 cases), although three received MenB vaccine and one a quadrivalent ACYW vaccine. For comparison, 499 controls (4.7%) had been vaccinated before the index date, 497 with MenC vaccine and two with MenB vaccine.

## Discussion

This study used the national French medico-administrative database to characterize risk factors for IMD in all individuals hospitalized for IMD in France over a recent six-year period (January 1st, 2012 to December 31st, 2017). Of the risk factors identified in European vaccination guidelines, congenital and acquired immunodeficiency, as well as asplenia/hyposplenia had the most robust associations with hospitalization for IMD. In addition, chronic medical conditions, not mentioned in the guidelines, such as autoimmune disorders, hemophilia, and severe chronic respiratory disorders were also strongly associated with hospitalization for IMD, as was a proxy measure of low socioeconomic status.

Overall, 3,532 cases of IMD were identified in the *SNDS* database, corresponding to around 600 incident cases per year. In France, IMD is a notifiable disease, and cases are monitored systematically by the French national public health surveillance agency (*Institut de Veille Sanitaire; Santé Publique*).^30^ Over the ten-year period from 2006 to 2015, 5,772 cases of IMD were notified (with an estimated under-reporting rate of 9%), again around 600 cases per year,^30^ suggesting that case identification in the present study using ICD-10 diagnostic codes in the *SNDS* database was accurate. The age distribution of the cases, with two peaks in infants ≤2 years old and in adolescents/young adults also matches the patterns in age-specific incidence rates in the national surveillance data.^30^

This study has a number of limitations. The most important are imputable to the nature of the SNDS database itself. For example, IMD cases were retrieved on the basis of the ICD-10 code on the hospital discharge summary. However, case reporting for epidemiological surveillance of IMD in France is based on a standardized definition based on composite bacteriological and clinical criteria.^30,31^ This specific information is not available in the *SNDS*, and it is not clear to what extent the two methods of documenting cases of IMD are consistent with each other. In addition, no information is available on certain risk factors, including some of those identified in European vaccination guidelines, which thus could not be assessed. This is the case for disease-related risk factors, such as the bacterial serogroup, and for biological variables such as measures of renal function. It would be of great interest to evaluate whether certain risk factors may be of particular relevance for individual Nm serogroups. Other medical risk factors may be underreported or reported under a more general term; for example, individuals with complement or properdin deficits may be documented under the more general term of congenital immunodeficiency disorders. Moreover, other risk factors of interest have been identified through proxy variables. This may contribute significant imprecision to the estimates, which should thus be interpreted with caution. These variables merit further investigation in dedicated studies. Social, environmental, and behavioral risk factors, which may be targets for prevention measures, cannot be quantified in the *SNDS*. We have attempted to identify low socioeconomic status through the proxy variables of *CMU-C* status and the SDI, but this is clearly only an indirect and partial approach to this important issue. Finally, certain high-risk social groups, such as migrants^32^ and the homeless, may not even be present in the *SNDS*, as well as patients who died before reaching hospital. Independently of the database itself, matching of controls to cases may not be perfect due to the presence of unidentified confounding variables. However, this potential source of error is expected to be of limited importance since we were, in principle, able to identify all known IMD cases in this study and because the source population for identifying controls corresponded to >99% of the French population. In any case, the approach used does not permit causality to be addressed for any of the associations observed.

On the other hand, the study has important strengths. Using a comprehensive medico-administrative database such as the *SNDS* enables virtually exhaustive identification of all IMD cases in the country over the dedicated study period of six years, using a standard definition. As a result, it was possible to collect over three thousand cases, providing robust statistical power to detect associations with potential risk factors. These sample sizes are difficult to achieve in case-control studies performed in populations enrolled from local outbreaks of IMD. In addition, notwithstanding the limitations mentioned above, the *SNDS* contains exhaustive data on a wide range of potential clinical risk factors documented in a standardized way, thus avoiding the problems of definition that may arise when multiple data sources are combined. Compared to surveillance monitoring data from *Santé Publique*, the *SNDS* offers comparable power for case detection associated with extensive information on comorbidities and risk factors that are not available from surveillance monitoring.

In our study, the age distribution of cases evolved over the inclusion period, with an increase in their median age from 19 to 25 years. This is most likely due to fluctuating secular trends in the Nm serogroups responsible for infection, with a relative decline over the period in the B serogroup, which is principally responsible for IMD in infants. This temporal trend has been accompanied by a rise in the number of infections due to the W and Y serogroups,^33^ which are more frequent in older (W serogroup) and younger (Y serogroup) adults.^12^ This evolution has been clearly documented in the data collected by the French national public health agency (*Santé Publique*).^30^ However, this hypothesis cannot be tested in the *SNDS* database since information on the serogroup is not documented in the database.

The data on vaccination status are difficult to interpret since no information on serogroup status of the IMD infection is available in the SNDS database. Nonetheless, the data indicate that, up until 2017, vaccination coverage was low, with <10% of cases and controls having been vaccinated. MenC vaccination has only been recommended in national vaccination guidelines in France since 2010 for one-year toddlers with a catch-up until the age of 24 years. MenACWY vaccination has been recommended since 2010 and MenB vaccination since 2014, both for at-risk populations and in specific circumstances such as local outbreaks. However, during the study period (2012–17), serogroup B represented more than half of notified IIM cases, ranging from 68.0% to 42.1%. The only year in which serogroup B represented <50% of cases was 2017. It would thus be expected that the majority of cases in our study represented serogroup B infections.

At-risk populations listed in most European vaccination recommendations include individuals with asplenia/hyposplenia (including sickle-cell disease), congenital immune disorders (such as complement or properdin deficits), and use of eculizumab. Although these events were extremely rare in both cases and controls (<0.1% overall), it was still possible to identify significant associations with the incidence of IMD.

Moreover, most of the other risk factors mentioned in the different European guidelines which could be extracted from the *SNDS* database showed significant associations with hospitalization for IMD. The association with HIV infections, which had been described previously in case series,^34,35^ was of a similar magnitude to that observed for asplenia. It was not possible to assess such associations for previous organ transplantation or for celiac disease due to insufficient numbers of cases. The most robust associations (those with the highest lower limit to the CI of the OR) were observed for congenital immunodeficiency as a class, acquired immunodeficiency/HIV infections and asplenia/hyposplenia.

Furthermore, a number of other risk factors were identified that are not mentioned in European vaccination guidelines. Some of these are relatively common in the general population and show a strong association with hospitalization for IMD. These include certain acute respiratory infections requiring hospitalization, prematurity, hemophilia, autoimmune diseases, severe chronic respiratory disorders and chronic bone marrow disorders. Several of these had been identified in a previous historical case series from the US in the last quarter of the twentieth century.^36^ From a public health perspective, certain high-risk groups identified here could also be considered for vaccination. Moreover, vaccination is currently targeted at children and young adults, whereas certain risk factors are much more common in older individuals, such as severe chronic respiratory diseases, certain autoimmune diseases, and renal failure. In this context, it is noteworthy that, although the number and incidence of IMD cases are much lower in individuals aged over 60 years compared to young children,^30^ the case fatality rate is around four times higher.^4^

Although recommendations already exist in most countries for vaccination of several at-risk groups (such as individuals with complement deficiencies or following splenectomy) against meningococcal diseases, this study has identified additional at-risk groups that are not consistently targeted by these recommendations, such as patients with HIV infections or patients with certain chronic or acute respiratory diseases. Outside the scope of general population vaccination programs, the present data may help inform decision making when considering vaccination of individuals in these additional at-risk groups.

The findings also strengthen the argument for harmonization of European vaccination guidelines,12 to include those medical risk factors which are either associated with the highest risk of IMD or which are particularly frequent. Nonetheless, the study was not intended to generate data to advocate a specific vaccination policy but rather to identify risk factors that may be associated with IMD. Assessing the interest of vaccinating target groups as a public health policy would require specific studies of the potential benefits in at-risk groups to be conducted, as well as health economic models to assess cost-effectiveness. The utility of anti-meningococcal vaccination in older patients would need to be evaluated in the context of potential immunosenescence. Feasibility, in terms of accessibility to at-risk individuals, to the opportunity to vaccinate, would have to be assessed, given the low uptake of non-mandatory vaccination programs in general.

In addition to these clinical risk factors, an association was also found between hospitalization for IMD and two markers of socioeconomic deprivation, the *CMU-C*, which is an individual marker of low income (which gives eligibility for comprehensive healthcare reimbursement in France) and the SDI, which is a composite ecological marker of social deprivation in the municipality of residence. Of the two, *CMU-C* was more strongly associated with hospitalization for IMD. Until children or young adults become financially independent, they benefit from the social security coverage of their parents, and *CMU-C* status is thus an indicator of low parental income. The association was the strongest for children of preschool age (≤4 years) when the home environment (for example, overcrowding) is probably the most important environmental risk factor for infection. This association disappeared in older children and in younger adults, perhaps indicating that the school or university environment becomes the principal environmental source of IMD risk. In financially independent adults, a higher risk of hospitalization for IMD was associated with their own *CMU-C* status and thus their own income. The present findings are consistent with numerous studies in different countries that have reported a higher frequency of IMD in low socioeconomic areas, and a lower risk of disease in children living in more favorable socioeconomic conditions (reviewed by Burgess in 2006).^37^ For example, two studies have demonstrated an association between ecological markers of social deprivation with incidence and mortality of IMD in urban and rural areas of England.^38,39^ It is thus important for social and health workers to ensure that vaccination recommendations are followed carefully for children living in socially deprived areas, where they might be at higher risk of exposure to infection.

In conclusion, this study has documented potential risk factors associated with hospitalization for IMD in a large and comprehensive sample of individuals with IMD in France. Several of the risk factors identified may help identify groups who could benefit from targeted prevention measures (such as vaccination) in order to reduce the burden of IMD. Moreover, the findings reinforce the importance for policymakers of targeting other patient groups who are at risk for IMD in order to ensure social equity in the face of this serious but preventable, disease.

## Supplementary Material

Supplemental Material

## Data Availability

GSK makes available anonymized individual participant data and associated documents from interventional clinical studies that evaluate medicines upon approval of proposals submitted to www.clinicalstudydatarequest.com. To access data for other types of GSK sponsored research, for study documents without patient-level data and for clinical studies not listed, please submit an inquiry via the website.
