# Supplementary material for: Risk factors for invasive meningococcal disease: a retrospective analysis of the French national public health insurance database
Source: Hum Vaccin Immunother. 2021 Jan 15;17(6):1858–66. doi: 10.1080/21645515.2020.1849518 (PMC8115611; doi:10.1080/21645515.2020.1849518)
Supplement: Supplemental Material [file KHVI_A_1849518_SM0911.zip › KHVI_1849518_Supplemental_Material.docx]

**SUPPLEMENTARY MATERIAL**

**Supplementary Table 1. National recommendations for meningitis vaccination of high-risk groups**

|  | **GERMANY**[**^21^**](#_ENREF_21) | **FRANCE**[**^22^**](#_ENREF_22) | **UNITED KINGDOM**[**^23^**](#_ENREF_23) | **ITALY**[**^24^**](#_ENREF_24) | **SPAIN**[**^25^**](#_ENREF_25) |
| --- | --- | --- | --- | --- | --- |
| Asplenia or hyposplenia (including sickle cell disease) | **×** | **×** | **×** | **×** | **×** |
| Immunosuppression (organ transplant, chemotherapy, high-dose corticosteroid use) |  |  |  | **×** |  |
| Congenital immunodeficiency | **×** |  |  | **×** |  |
| Human immunodeficiency virus infection |  |  |  | **×** | **×** |
| Acquired immunodeficiency | **×** |  |  |  |  |
| Complement (C5 –C9) deficiency | **×** | **×** | **×** | **×** | **×** |
| Hematopoeietic stem cell transplantation |  | **×** |  |  | **×** |
| Type 4 toll-like receptor deficiency |  |  |  | **×** |  |
| Properdin deficiency | **×** | **×** |  | **×** |  |
| Eculizumab therapy | **×** | **×** |  |  | **×** |
| Hypo γ-globulinemia | **×** |  |  |  |  |
| Renal insufficiency with creatinine clearance <30 ml/min |  |  |  | **×** |  |
| Serious chronic liver disease |  |  |  | **×** |  |
| Type 1 diabetes mellitus |  |  |  | **×** |  |
| Splenic dysfunction due to celiac disease |  |  | **×** |  |  |
| Loss of cerebrospinal fluid |  |  |  |  |  |

**×**: Risk factor mentioned in guidelines

Shaded boxes: risk factors assessed in this study.

**Supplementary Table 2. Database codes and algorithms used to identify risk factors for invasive meningococcal disease**

| Congenital immunodeficiency | ICD-10 codes D800, D801, D803, D805, D806, D808, D809, D818, D819, D830 to D832, D838, D839, D841, D848 or D849 |
| --- | --- |
| Complement deficiency | D841 |
| Eculizimab therapy | ATC code L04AA25 |
| Hematopoietic stem cell transplantation | ICD-10 codes Z94800, Z94801 or Z94802  OR DRG codes 27Z021, 27Z022, 27Z023, 27Z024, 27Z03Z or 27Z04J |
| Acquired immunodeficiency | ALD 7 |
| HIV infection | ICD-10 codes B20 to B24 |
| Asplenia or hyposplenia | ICD-10 codes D730, D735, K900, K703, D57, Q893, E310, E752, E85 or D561  OR CCAM codes FFFA001, FFFA002, FFFC001 or FFFC420 |
| Sickle cell disease | D57 |
| Serious chronic liver disease | ALD 6 |
| Type 1 diabetes mellitus | ALD 8 + algorithm to distinguish Type 1 and 2 DM* |
| Celiac disease | ICD-10 code K900 |
| Organ transplant | ALD 28 |
| Cancer | ALD 30 |
| Renal disease | ICD-10 codes N00 to N08, N10 to N19, N25 or Z49  OR DRG codes 11K02, 11M06, 11M08, 28Z01, 28Z02, 28Z03, 28Z04 |
| Prematurity | ICD-10 code P07 |
| Acute upper respiratory tract infections | ICD-10 codes J00 to J006 |
| Acute lower respiratory tract infections | ICD-10 codes J10 to J15, J17, J18, J20 or J21 |
| Bronchitis | ICD-10 code J20 |
| Pneumopathy | ICD-10 codes J14, J15, J17 or J18 |
| Influenza | ICD-10 codes J09 to J11 |
| Bronchiolitis or HRSV infections | ICD-10 code J21 |
| Autoimmune diseases | ALD 21, 22 or 25 |
| Hemophilia | ALD 11 |
| Severe chronic respiratory disorders | ALD 14 |
| Chronic bone marrow disorders | ALD 2 |
| Paraplegia | ALD 20 |
| Incapacitating stroke | ALD 1 |
| Psychosis | ALD 23 |
| Chronic neurological disorders | ALD 9 |
| Cardiac failure | ALD 5 |
| Atherosclerosis | ALD 13 |
| Type 2 diabetes mellitus | ALD 8 + algorithm to distinguish Type 1 and 2 DM* |

**Supplementary Table 3. Chronic diseases giving eligibility for full healthcare reimbursement in France (*ALD* status)**

| 1. | Invalidating stroke |
| --- | --- |
| 2. | Bone marrow deficiency and other chronic cytopenias |
| 3. | Chronic arterial disease with ischemic manifestations |
| 4. | Complicated bilharzia |
| 5. | Severe heart failure, severe cardiac rhythm disorder, serious valvulopathies, severe congenital cardiopathies |
| 6. | Chronic active liver disease, cirrhosis |
| 7. | Severe primary immunodeficiency necessitating long-term treatment, human immunodeficiency virus infection |
| 8. | Type 1 or Type 2 diabetes mellitus |
| 9. | Severe neurological and muscular disorders (including myopathies), serious epilepsy |
| 10. | Severe congenital or acquired hemoglobinopathies or hemolytic disorders |
| 11. | Hemophilia and serious congenital hemostatic disorders |
| 13. | Coronary disease |
| 14. | Severe chronic respiratory failure |
| 15. | Alzheimer’s disease, other dementias |
| 16. | Parkinson’s disease |
| 17. | Hereditary metabolic disorders necessitating long-term specialized treatment |
| 18. | Cystic fibrosis |
| 19. | Severe chronic kidney disease and primary nephrotic syndrome |
| 20. | Paraplegia |
| 21. | Vasculitis, systemic lupus erythematosus, systemic scleroderma |
| 22. | Progressive rheumatoid arthritis |
| 23. | Long-term psychiatric disorders |
| 24. | Progressive ulcerative colitis and Crohn’s disease |
| 25. | Multiple sclerosis |
| 26. | Progressive structural idiopathic scoliosis |
| 27. | Severe spondylarthritis |
| 28. | Consequences of organ transplantation |
| 29. | Active tuberculosis, leprosy |
| 30. | Malignant tumor, malignant disease of the hematopoietic or lymphatic tissue |

*ALD*: *affection de longue durée* (long-term disease status); *CMU-C*: *couverture maladie universelle complémentaire* (proxy variable for low income).

Supplementary Table 4. Description of cases of invasive meningococcal disease according to age group and year of hospitalization

| **Year** | **Number of cases** | | | | **Age** | |
| --- | --- | --- | --- | --- | --- | --- |
|  | <25 years | 25-59 years | ≥60 years | All ages | Mean ± SD | Median |
| 2012 | 389 | 145 | 108 | 642 | 27.4 ± 26.5 | 19 |
| 2013 | 393 | 163 | 125 | 681 | 28.3 ± 26.9 | 20 |
| 2014 | 281 | 132 | 88 | 501 | 27.9 ± 26.5 | 21 |
| 2015 | 310 | 130 | 106 | 546 | 29.8 ± 27.6 | 19 |
| 2016 | 309 | 157 | 111 | 577 | 31.1 ± 28.3 | 22 |
| 2017 | 288 | 158 | 139 | 585 | 33.8 ± 29.2 | 25 |
| **TOTAL** | **1970** | **885** | **677** | **3532** | **29.7 ± 27.6** | **21** |

SD: standard deviation.
