# Supplementary figures and images for: Risk factors for invasive meningococcal disease: a retrospective analysis of the French national public health insurance database
Source: Hum Vaccin Immunother. 2021 Jan 15;17(6):1858–66. doi: 10.1080/21645515.2020.1849518 (PMC8115611; doi:10.1080/21645515.2020.1849518)

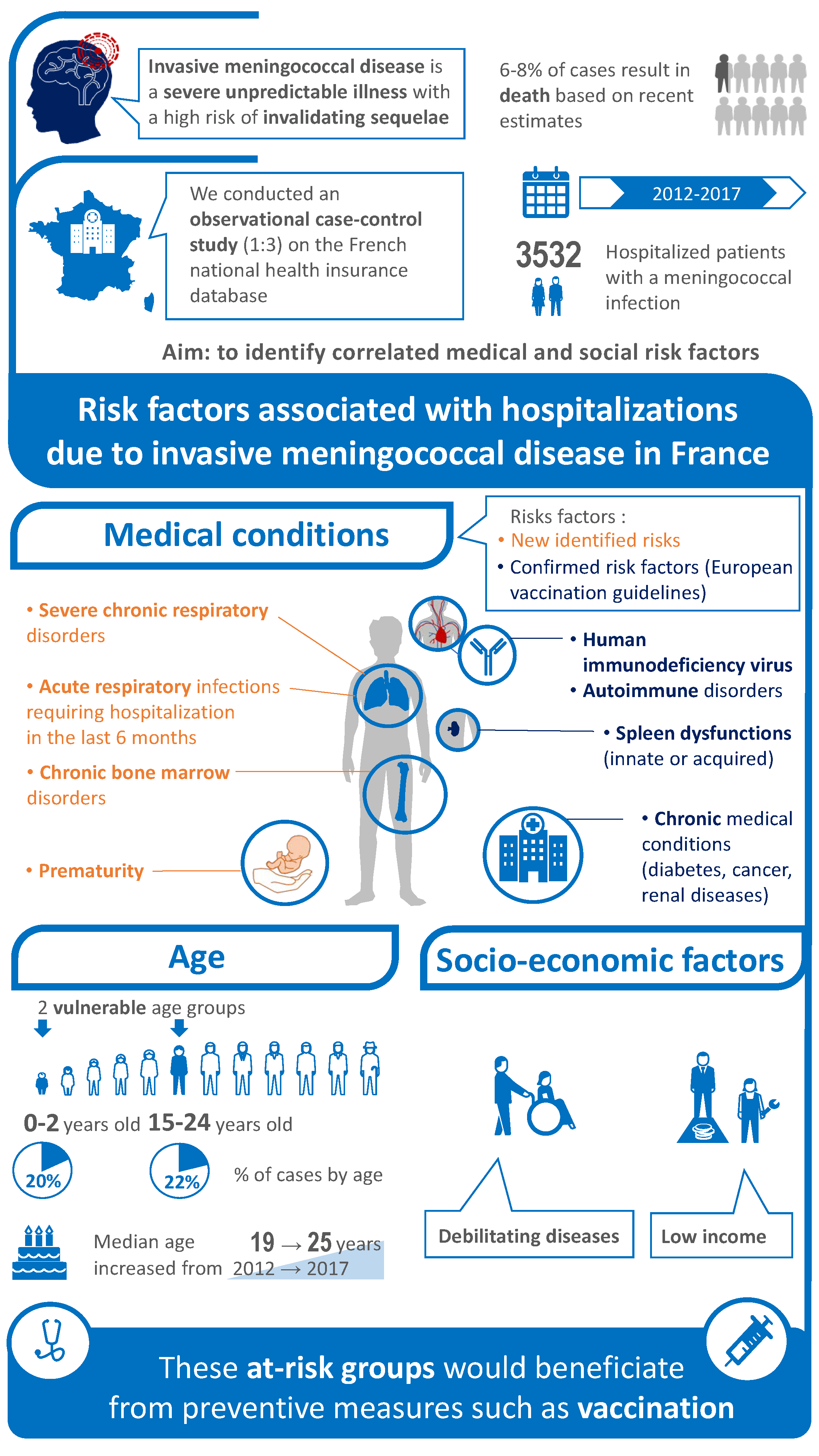

Supplement: Supplemental Material [file KHVI_A_1849518_SM0911.zip › KHVI_1849518_Graphical Summary.tif]
